# Purification for Carbon Nanotubes Synthesized by Flame Fragments Deposition via Hydrogen Peroxide and Acetone

**DOI:** 10.3390/ma13102342

**Published:** 2020-05-20

**Authors:** Asmaa H. Hammadi, Ahmed M. Jasim, Firas H. Abdulrazzak, Abdulkareem M. A. Al-Sammarraie, Yacine Cherifi, Rabah Boukherroub, Falah H. Hussein

**Affiliations:** 1 College of Pharmacy, University of Babylon, Hilla 51002, Iraq; phar.asmaa.hashim@uobabylon.edu.iq; 2 Department of Biomedical, Biological and Chemical Engineering, University of Missouri, Columbia, MO 65211, USA; amjtg6@mail.missouri.edu; 3 Chemistry Department, College of Education for Pure Sciences, Diyala University, Baqubah 32001, Iraq; firas.habeeb@puresci.uodiyala.edu.iq; 4 Department of Chemistry, College of Science, University of Baghdad, Baghdad 10070, Iraq; karim.alsamuraee@uobaghdad.edu.iq; 5 Univ. Lille, CNRS, Centrale Lille, ISEN, Univ. Valenciennes, UMR 8520-IEMN, F-59000 Lille, France; yacine.dz15@gmail.com (Y.C.); rabah.boukherroub@iemn.univ-lille1.fr (R.B.); 6 Pharmacy Department, Al-Mustaqbal University College, Babylon 51002, Iraq

**Keywords:** flame fragments deposition, hydrogen peroxide, liquefied petroleum gas, CNTs purification

## Abstract

Carbon nanotubes (CNTs) are synthesized by the flame fragment deposition (FFD) technique using Iraqi liquefied petroleum gas (LPG) as a source of carbon in a hand-made reactor at a low temperature (160 °C) without using a catalyst. Purification of the multi-walled carbon nanotubes (MWCNTs) is carried out using a two-step process consisting of sonication in 30 wt.% hydrogen peroxide (H_2_O_2_) solution at room temperature to remove amorphous impurities adhering to the walls of the CNTs and carbon nanoparticles (CNPs), followed by sonication in an acetone bath to remove the polyaromatic hydrocarbons (PAH) formed during the LPG gas burning. Comprehensive characterizations such as X-ray diffraction (XRD), atomic force microscopy (AFM), thermo-gravimetric analysis (TGA), and transmission electron microscopy (TEM) were conducted to verify the efficiency of the purification process. The results clearly demonstrated that this process is promising for the purification of the synthesized CNTs.

## 1. Introduction

Since their discovery in 1990 [1], carbon nanotubes (CNTs) have attracted remarkable interest because of their unique physical [2], chemical [3], and mechanical properties [4,5]. They have been used in various applications such as catalysis [6], biotechnology and biomedicine [7,8], probing of field emission stability [9], and even as a template to make some metal oxide nanostructures [10]. Upon the synthesis processes, the fresh surface has a lot of impurities such as amorphous carbon (non-crystallized), catalyst metals, and so on. Since most of the CNTs modern application depends on their purity, several procedures have been developed for their purification. The purification techniques are classified to three main methods, namely physical, chemical, and the combination of chemical and physical methods [11]. All the purification methods deal with one target, which is the removal of amorphous carbon, carbon nanoparticles, and the residual catalyst that was used in CNTs synthesis [12,13,14]. Each technique presents one or more disadvantages such as non-selectivity, damage of nanotubes, formation of toxic wastes, and time consumption. The physical methods such as ultrasonication, filtration, and centrifugation are considered as mild and less effective methods, however, they do not destroy the pristine structure [15,16,17]. In chemical purification, there are two routes including dry and wet conditions. The dry refers to oxidation under air [18], oxygen, and other gases [19], while the wet conditions refer to using of strong liquid oxidants. Martinez et al. [20] purified SWNTs by a combination of air treatment/microwave, leading to a high metal removal within a short time as compared with acid reflux treatments. Gomez et al. [21] used a method for purification that consists of two steps. The first one is treatment in a microwave and the second step consists of a gas-phase chlorination. Piranha mixtures whether the acidic solution (H_2_SO_4_/H_2_O_2_) or the alkaline solution (NH_4_OH/H_2_O_2_) [22] are used in the wet oxidation. The CNTs purification in nitric acid was also reported [23,24]. The nitric acid purification results in an effective purification as they able to unzip the tube wall removing the amorphous carbon as well as the metal catalyst. However, it is destructive and could damage the pristine structure of the CNTs backbone. Other people have found potassium permanganate (KMnO_4_) to be an effective oxidant [25]. Colomer et al. [26] have reported an effective oxidation using KMnO_4_ by applying a low temperature of 80 °C and found, through transmission electron microscopy (TEM) imaging, all the amorphous aggregates were removed. Unfortunately, when KMnO_4_ is used, further steps are required such as a long washing time to remove the formed MnO_2_ [27].

On the other hand, hydrogen peroxide H_2_O_2_ was found to be a promising oxidant that is capable to attack the carbon surface without destroying the pristine structure of the CNTs, requiring less washing times, and not to mention that it produces water as a by-product [28]. Rasel et al. [29] investigated other mixtures of chemical agents for purifying MWNTs such as a mixture of hydrogen peroxide with KOH or HCl. HCl/H_2_O_2_ showed 100% purification as compared to HCl and KOH/H_2_O_2_ with purification yields of 93.46% and 3.92%, respectively. Nevertheless, the use of H_2_O_2_ solely at a low concentration lacks the capability to remove metal traces embedded in the CNTs structure wall. Suzuki et al. [30] have reported an efficient purification method of arc-plasma-single wall carbon nanotubes (SWCNT) by reflux in a H_2_O_2_ solution in the presence of iron particles as a catalyst. Their results showed a 90% purification by removing all the amorphous carbon material. They attributed that to the active oxygen that was created by the reaction between iron and H_2_O_2_.

Herein, we have synthesized carbon nanotubes with no need for a catalyst. Thus, H_2_O_2_ oxidation could perfectly fit. It was followed by a purification in acetone to ensure dissolving any left-over organic residues originated from the used LPG gas. This process was compared with only H_2_O_2_. We observed the influence of both oxidation techniques on thermal stability by using thermogravimetric analysis (TGA). The oxidation by H_2_O_2_ followed by acetone washing method showed a higher thermal stability of the CNTs than those purified only through oxidation with H_2_O_2_. The changes in the morphology were monitored using transmission electron microscopy (TEM) and atomic force microscopy (AFM). The structure was further examined by powder X-ray diffraction (XRD). We envision that the sequential purification with hydrogen peroxide and acetone could provide an effective, environmentally friendly, and less expensive technique.

## 2. Materials and Methods

### 2.1. Materials

Iraqi liquefied petroleum gas (LPG) was purchased from the local market in Babylon Governorate. The materials used in this experiment was methanol (99.85%) from Hyman, England, acetone (99%) from S.D. Fine-chem. Ltd., City, India and hydrogen peroxide (30% H_2_O_2_) from Barcelona, Scharlab Spain. The N_2_ (99.999%) gas was purchased from Emirates industrial gases, Dubai, United Arab Emirates. The standard MWNTs, used for comparison with synthesized CNTs in this study with a purity of 95% and mode diameter of 5.5 nm were purchased from Aldrich. All materials were used without further purification.

### 2.2. Carbon Nanotubes Synthesis

**Figure 1:**
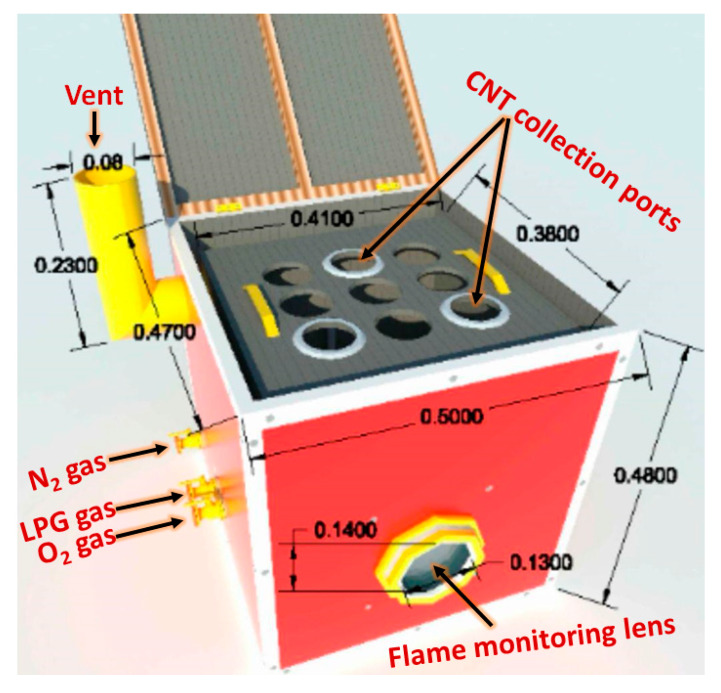
The homemade flame fragments deposition (FFD) instrument.

The Flame Fragments Deposition (FFD) process was performed using a homemade chamber instrument constructed for the synthesis of carbon nanotubes (CNTs) from LPG as the carbon source [31]. The homemade FFD instrument consists of two stainless steel boxes. The internal box (41 × 38 × 25 cm^3^), LPG, oxygen, and nitrogen gases were connected to the system through three stainless inlet tubes with a diameter of 1.5 cm. The flow rate of gases was controlled by outside gauges. The instrument consists of a gas gate for the excess gases. On the top of the box, there are nine positions fitted to hold the crucibles used as sample collectors. These collectors are in touch with the cold upper lid. The outer box was designed with dimensions of 50 × 47 × 38 cm^3^. The outer box has an airtight cover. The instrument has a front lens with dimensions of 14 × 13 cm^2^, used to monitor the intensity of the flame. The schematic diagram of the homemade flame fragments deposition instrument is shown in Figure 1.

### 2.3. Purification Processes

Two routes were used for the purification of the synthesized CNTs. The first is an oxidation with H_2_O_2_, while the second one consists of an oxidation with H_2_O_2_ followed by acetone treatment using a separation funnel. 100 mg of as-prepared CNTs were dispersed with an ultrasonic water bath in 50 mL of H_2_O_2_ for one hour. The mixture was left in a refrigerator at 4 °C for 24 h, after that, the solution allowed to reach room temperature then heated gradually to 50 °C until all hydrogen peroxide was completely destroyed. Finally, the sample was washed with deionized water and dried at 80 °C for 4 h. The second route comprises the same steps with an additional washing step with acetone of the dried sample. The treatment with acetone includes dispersion of CNTs in 15 mL of acetone and sonication for 15 min. The suspension was then centrifuged for 15 min. The separated CNTs were then calcined at 275 °C for 2 h.

### 2.4. Characterizations

The X-ray diffraction (XRD) patterns were performed on a (RU-200B) (Rigaku, Tokyo, Japan) X-ray diffractometer using Cu Kα radiation (wavelength = 0.15405 nm) with a Ni filter. The tube current was 100 mA with a tube voltage of 40 kV. The *2θ* angular region between 10 and 80° was explored at a scan rate of 5 °/min. For all the XRD analysis, the resolution in the *2θ* scans was kept at 0.02°. Initial transmission electron microscopy (TEM) measurements to confirm morphology were performed using JEOL JEM-1400 (Jeol Inc., Peabody, MA, USA) equipped with Lanthanum-hexaboride (LaB_6_) filament and operated at an acceleration voltage 120 kV and data were collected on the Gatan Ultrascan 1000 CCD camera (Thermo Fisher Scientific, Hillsboro, OR, USA). Subsequent measurements were performed using FEI Tecnai F30 Twin microscope (Thermo Fisher Scientific, Hillsboro, OR, USA) equipped with analytical spectrometers (Bruker Quantax 400 Silicon Drift Detector or Quantum 963 Gatan Image filter (GIF)) to get high magnified TEM images. Atomic Force Microscopy (AFM) was performed by placing a well-dispersed droplet of CNTs ink using (SPM-A3000, Angstrom Advanced Inc., Stoughton, MA, USA, Atomic force microscopy (AFM)-Contact mode).

## 3. Results and Discussion

**Figure 2:**
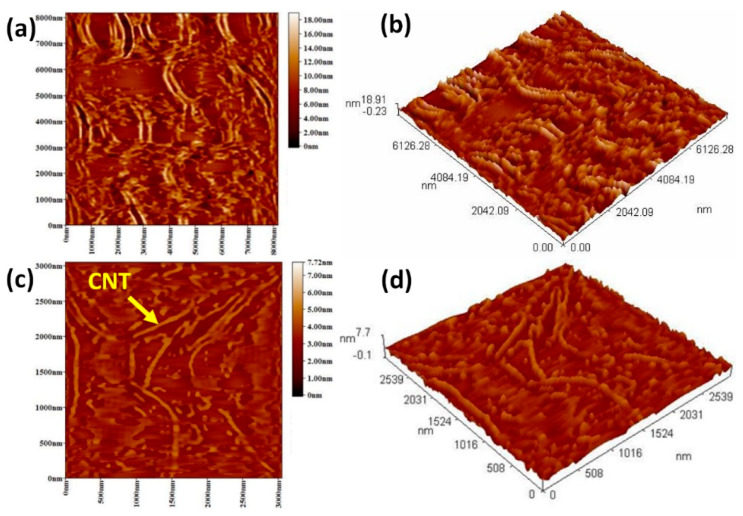
Atomic force microscopy (AFM) images of produced MWNTs: (**a**,**b**) 2-D and 3-D sections before any treatment, (**c**,**d**) 2-D and 3-D sections after treatment by hydrogen peroxide and acetone.

Atomic force microscopy (AFM) is highly powerful tool [32] with a high resolution less than a few nanometers. Therefore, using such tool provides us a sense on how the material topography is distributed. In Figure 2, the AFM image shows the samples in 2-D and 3-D before and after treatment in hydrogen peroxide and acetone. Figure 2a,b depicts the material as bundles or quaffs of carbon filaments consisting of minimal fraction CNTs. Many aggregations are present resulting from remaining particles on the surface. The existence of heavy oil materials as part of LPG, which form agglomerates can be considered as the reason behind such morphology. Even though there are some CNTs, their hydrophobic surface nature leads to their agglomeration through Van der Waals forces [33]. After treating the above product with hydrogen peroxide and acetone in a sequential process, the AFM shows a tubular structure with a pattern of bundles as in Figure 2c,d. It is important to mention that H_2_O_2_ solely failed to decompose the oil materials. Nevertheless, the synthesized CNTs after sequential treatment with H_2_O_2_ followed by acetone has shown a descent evidence of having CNTs structure with a length of 0.8–2.1 μm with a tubular structure.

**Figure 3:**
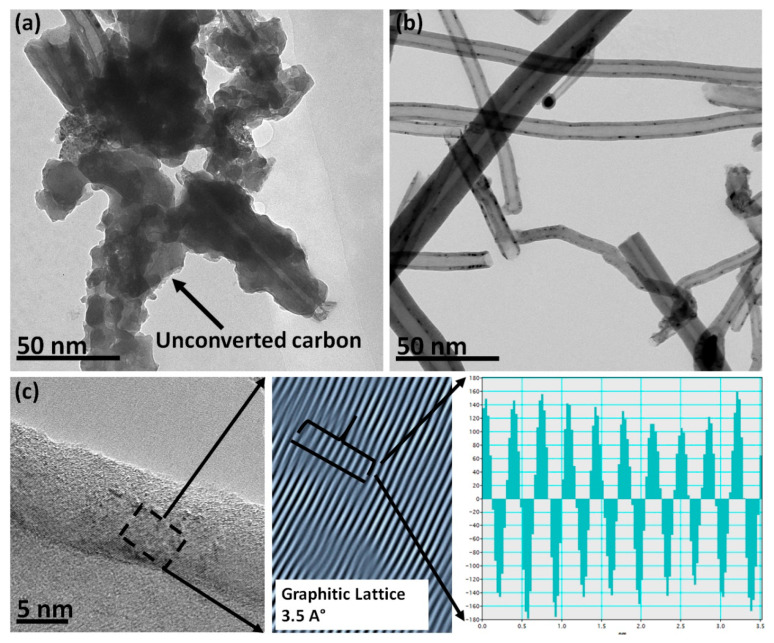
Transmission electron microscopy (TEM) images: (**a**) before treatment, (**b**) after treatment with H_2_O_2_ and acetone, and (**c**) high-resolution images with graphite inner plans (lattices masked by Gatan Software 3.0).

Figure 3a displays a low-resolution transmission electron microscopy (TEM) image of synthesized CNTs before any purification. It can be seen that the unconverted carbon is wrapping the CNTs bundles. However, these impurities were removed after the CNTs exposure to H_2_O_2_ then acetone. Figure 3b depicts the TEM image of the CNTs after treatment with H_2_O_2_ and acetone. Hydrogen peroxide removes the impurities without damaging the CNTs or forming any byproduct such salts, which may adsorb on the inner or outer part of the tubular structure. The influence of H_2_O_2_ is not only limited to purification but can also serve to functionalize the outer surface of CNTs. The functionalization process of the CNTs surface produces C–O, C=O, and C–OH groups, which reduces Van der Walls forces between CNTs bundles, allowing to visualize the CNTs as single filaments.

**Figure 4:**
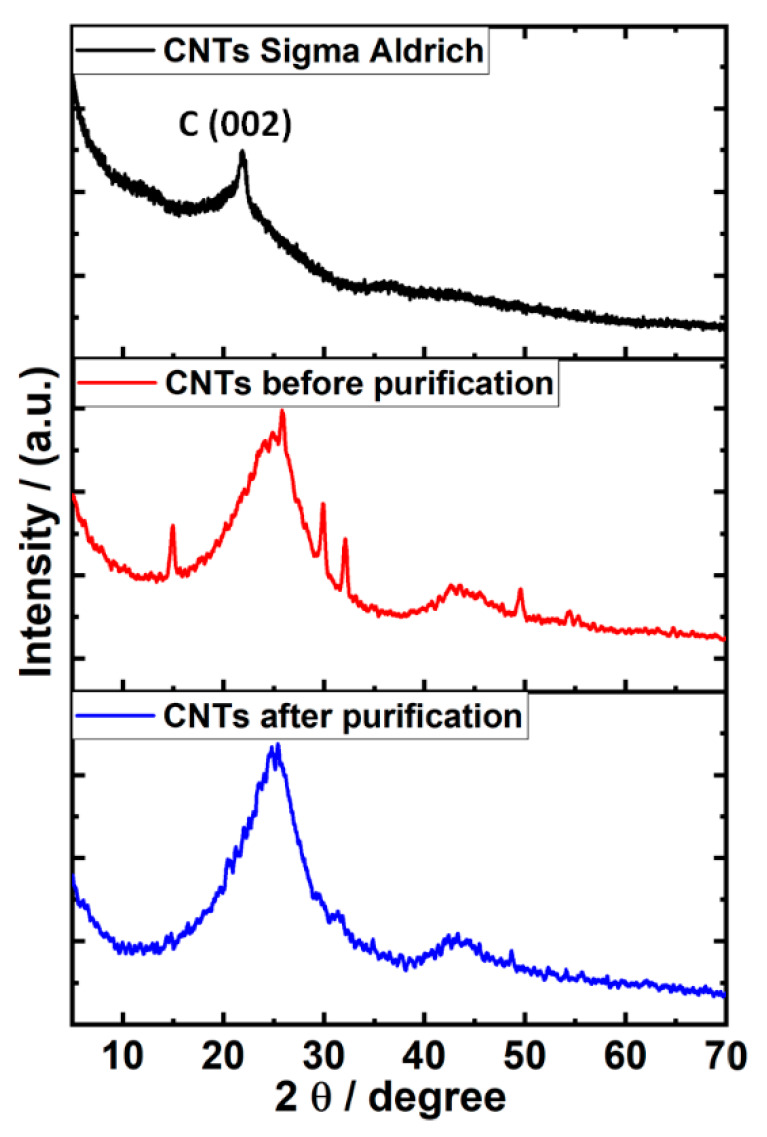
X-ray diffraction (XRD) patterns of multi-walled carbon nanotubes (MWCNTs) from Aldrich and the synthesized carbon nanotubes (CNTs) after purification with H_2_O_2_/acetone.

For further investigation, high-resolution imaging was conducted to check the surface structure. Figure 3c depicts a TEM image at an acceleration voltage of 200 kV showing the graphite lattice interplanar spacing of 0.35 nm (as in the inset). The high-resolution image is highly crucial since it provides information on the synthesized material whether it is CNTs or CNFs. We observed a high crystallinity on the outer wall with a thin film of amorphous film. This indicates that the synthesized material is neither fully crystalline nor amorphous. To resolve such ambiguity, X-ray diffraction (XRD) was recorded to gain more insights. Figure 4 displays the XRD pattern of MWNTs obtained from Aldrich for comparison purpose as a benchmark. It exhibits two characteristic peaks at 2θ = 26.32° and 43.46° assigned to the C (100) and C (002) planes of the carbon nanotubes, respectively [34]. The synthesized CNTs show similar peaks at 2θ = 26.56° and 44.16° after treatment with H_2_O_2_/acetone. However, impurities appear in the sample before treatment with H_2_O_2_/acetone with four distinguished peaks at 2θ = 14.78°, 29.47°, 31.86°, and 49.38°. The peaks at 2θ = 14.78° and 29.47° can be assigned to remaining and unconverted carbon, while the second two peaks refer to aluminum used as support for precipitation [35]. Regarding the surface graphitizing degree, all samples show peaks, indicating that crystalline structure planes are present.

**Figure 5:**
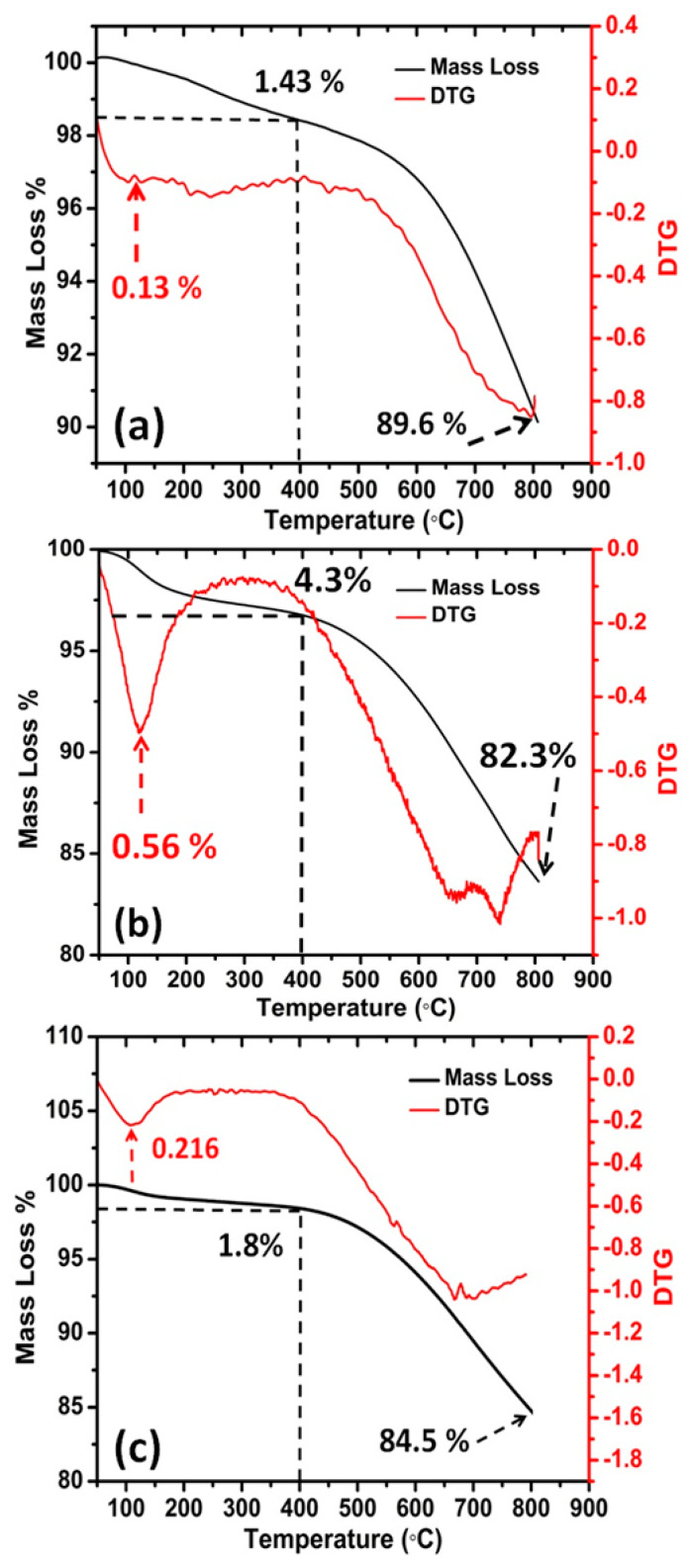
Thermo-gravimetric analysis (TGA) thermograms of: (**a**) MWNTs (Aldrich), (**b**) CNTs treated with hydrogen peroxide, and (**c**) treated with hydrogen peroxide then acetone.

Thermo-gravimetric analysis (TGA) is commonly used to assess the purity and quality of the synthesized CNTs, because the support and support/catalyst influence directly CNTs purity and type. The temperature at which a carbon material is oxidized is an indication of its stability. Typically, it is used to obtain a semi-quantitative estimation of the fraction of CNT in a sample, but it is not a precise quantitative technique. Figure 5a shows the TGA thermogram recorded under nitrogen gas for MWNTs from Aldrich, which reveals that the mass loss occurs in two steps. At 200 °C < T < 400 °C, a first mass loss of about 1.43% that can be attributed to the surface adsorbed water which is supported by the endothermic peak, as seen in derivative temperature curve. Above 400 °C, about 8.97% loss takes place related to some left-over amorphous material attached to graphic structure of MWNTs.

Figure 5b represents the thermal analysis of the prepared CNTs sample after treatment with only H_2_O_2_. It shows two steps: at T < 400 °C, there is a mass loss of about 4.3% that can be attributed to the evaporation of water and heavy organic material introduced through the deposition process [33]. In the 400–800 °C temperature range, a mass loss of 13.4% was observed due to the decomposition of more organic compounds on the CNTs such as amorphous carbon or traces of PAH residues.

Figure 5c exhibits the TGA thermogram of CNTs after treatment with H_2_O_2_/acetone, revealing 1.8% mass loss up to 400 °C, which is slightly lower than the mass loss recorded for CNTs after treatment with H_2_O_2_. Most of the mass loss is initiated at 390 °C and equals to 14.03%. In total, the sample lost 15.47 wt.% from heating to 800 °C and the ratio of CNTs was 84.53%. The difference of the thermal behavior of CNTs treated with H_2_O_2_ and H_2_O_2_/acetone in the 30–400 °C temperature range may be due to successful removal of oils materials by acetone.

## 4. Conclusions

In this work, we have demonstrated that flame fragments deposition (FFD) method using Iraqi LPG as a source of carbon in a hand-made reactor at low temperature (160 °C) without using a catalyst can be successfully applied for the synthesis of carbon nanotubes. The obtained crude CNTs are covered with amorphous carbon and polycyclic aromatic hydrocarbons. Two chemical protocols have been tested to purify the crude CNTs, consisting of hydrogen peroxide (H_2_O_2_) or H_2_O_2_/acetone treatment. While H_2_O_2_ treatment allows one to remove the amorphous carbon layer, H_2_O_2_/acetone turned out to be a good method for the purification of synthesized CNTs, exhibiting the higher thermal stability when eliminating the interference materials. TEM reflected carbon quantum dots decorated CNTs, which extends their properties for new advanced applications.
